# Supplementary material for: Gcorn fungi: A Web Tool for Detecting Biases between Gene Evolution and Speciation in Fungi
Source: J Fungi (Basel). 2021 Nov 12;7(11):959. doi: 10.3390/jof7110959 (PMC8624827; doi:10.3390/jof7110959)
Supplement: Supplementary file 1 [file jof-07-00959-s001.zip › FigureS1.pdf]

Gcorn fungi

Database of fungi gene phylogeny

★ Query & result

Query	stress
Taxon	*Agaricus bisporus* var. *bisporus H97*
Hits	19

★ Candidate

Gcorn	RefSeq	Gene product	Species	UniProt	Identifiers	CDD	Gene annotation
Homology Orthology	XP_006463693.1	hypothetical protein	*Agaricus bisporus var. bisporus H97*	Not available	AGABI2DRAFT_120501	107221	cd06464; Alpha-crystallin domain (ACD) of alpha-crystallin-type small(s) heat shock proteins (Hsps). sHsps are small stress induced proteins with monomeric masses between 12 -43 kDa, whose common feature is the Alpha-crystallin domain (ACD). sHsps are generally...
Homology Orthology	XP_006461924.1	hypothetical protein	*Agaricus bisporus var. bisporus H97*	Not available	AGABI2DRAFT_205955	107221, 223149	Alpha-crystallin domain (ACD) of alpha-crystallin-type small(s) heat shock proteins (Hsps). sHsps are small stress induced proteins with monomeric masses between 12 -43 kDa, whose common feature is the Alpha-crystallin domain (ACD). sHsps are generally...; similar to predicted protein; Molecular chaperone IbpA, HSP20 family [Posttranslational modification, protein turnover, chaperones]; COG0071; putative dimer interface [polypeptide binding]; cd06464
Homology Orthology	XP_006459441.1	hypothetical protein	*Agaricus bisporus var. bisporus H97*	Not available	AGABI2DRAFT_191417	107221	Alpha-crystallin domain (ACD) of alpha-crystallin-type small(s) heat shock proteins (Hsps). sHsps are small stress induced proteins with monomeric masses between 12 -43 kDa, whose common feature is the Alpha-crystallin domain (ACD). sHsps are generally...; similar to predicted protein; cd06464
Homology Orthology	XP_006457705.1	Skn7 response regulator receiver	*Agaricus bisporus var. bisporus H97*	Not available	AGABI2DRAFT_198669; SKN7; pfam00447	304365, 278854	Skn7 acts as a transcription factor involved in cellular responses to oxidative stress; HSF-type DNA-binding; cl21462; Basic leucine zipper (bZIP) domain of bZIP transcription factors: a DNA-binding and dimerization domain
