# Supplementary material for: Gcorn fungi: A Web Tool for Detecting Biases between Gene Evolution and Speciation in Fungi
Source: J Fungi (Basel). 2021 Nov 12;7(11):959. doi: 10.3390/jof7110959 (PMC8624827; doi:10.3390/jof7110959)
Supplement: Supplementary file 1 [file jof-07-00959-s001.zip › FigureS4bc.pdf]

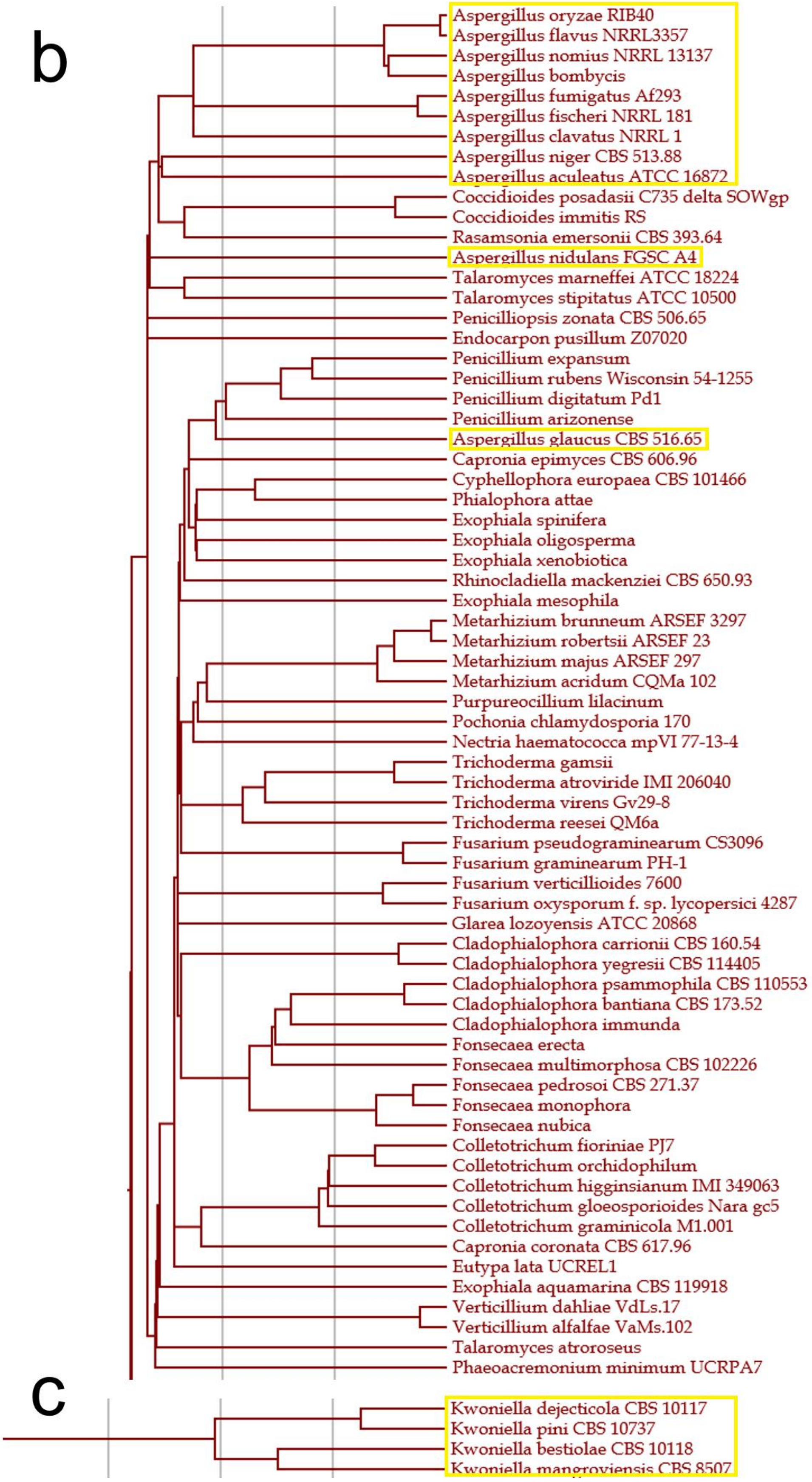
b
Aspergillus oryzae RIB40
Aspergillus flavus NRRL3357
Aspergillus nomius NRRL 13137
Aspergillus bombycis
Aspergillus fumigatus Af293
Aspergillus fischeri NRRL 181
Aspergillus clavatus NRRL 1
Aspergillus niger CBS 513.88
Aspergillus aculeatus ATCC 16872
Coccidioides posadasii C735 delta SOWgp
Coccidioides immitis RS
Rasamsonia emersonii CBS 393.64
Aspergillus nidulans FGSC A4
Talaromyces marneffei ATCC 18224
Talaromyces stipitatus ATCC 10500
Penicilliopsis zonata CBS 506.65
Endocarpon pusillum Z07020
Penicillium expansum
Penicillium rubens Wisconsin 54-1255
Penicillium digitatum Pd1
Penicillium arizonense
Aspergillus glaucus CBS 516.65
Capronia epimyces CBS 606.96
Cyphellophora europaea CBS 101466
Phialophora attae
Exophiala spinifera
Exophiala oligosperma
Exophiala xenobiotica
Rhinocladiella mackenziei CBS 650.93
Exophiala mesophila
Metarhizium brunneum ARSEF 3297
Metarhizium robertsii ARSEF 23
Metarhizium majus ARSEF 297
Metarhizium acridum CQMa 102
Purpureocillium lilacinum
Pochonia chlamydosporia 170
Nectria haematococca mpVI 77-13-4
Trichoderma gamsii
Trichoderma atroviride IMI 206040
Trichoderma virens Gv29-8
Trichoderma reesei QM6a
Fusarium pseudograminearum CS3096
Fusarium graminearum PH-1
Fusarium verticillioides 7600
Fusarium oxysporum f. sp. lycopersici 4287
Glarea lozoyensis ATCC 20868
Cladophialophora carrionii CBS 160.54
Cladophialophora yegresii CBS 114405
Cladophialophora psammophila CBS 110553
Cladophialophora bantiana CBS 173.52
Cladophialophora immunda
Fonsecaea erecta
Fonsecaea multimorphosa CBS 102226
Fonsecaea pedrosoi CBS 271.37
Fonsecaea monophora
Fonsecaea nubica
Colletotrichum fioriniae PJ7
Colletotrichum orchidophilum
Colletotrichum higginsianum IMI 349063
Colletotrichum gloeosporioides Nara gc5
Colletotrichum graminicola M1.001
Capronia coronata CBS 617.96
Eutypa lata UCREL1
Exophiala aquamarina CBS 119918
Verticillium dahliae VdLs.17
Verticillium alfalfae VaMs.102
Talaromyces atroroseus
Phaeoacremonium minimum UCRPA7
c
Kwoniella dejecticola CBS 10117
Kwoniella pini CBS 10737
Kwoniella bestiolae CBS 10118
Kwoniella mangroviensis CBS 8507
