# Supplementary material for: Gcorn fungi: A Web Tool for Detecting Biases between Gene Evolution and Speciation in Fungi
Source: J Fungi (Basel). 2021 Nov 12;7(11):959. doi: 10.3390/jof7110959 (PMC8624827; doi:10.3390/jof7110959)
Supplement: Supplementary file 1 [file jof-07-00959-s001.zip › TableS1.pdf]

Supplementary Table S1. Summary of fungi used for *Gcorn fungi*.

Taxonomy name	Genes
Agaricus bisporus var. *bisporus* H97	10448
Agaricus bisporus var. *burnettii* JB137-S8	11278
Alternaria alternata	13466
Anthracocystis flocculosa PF-1	6877
Arthrobotrys oligospora ATCC 24927	11479
Arthroderma otae CBS 113480	8765
Ascoidea rubescens DSM 1968	6787
Aspergillus aculeatus ATCC 16872	10843
Aspergillus bombycis	12263
Aspergillus clavatus NRRL 1	9121
Aspergillus fischeri NRRL 181	10395
Aspergillus flavus NRRL3357	13485
Aspergillus fumigatus Af293	9630
Aspergillus glaucus CBS 516.65	11255
Aspergillus nidulans FGSC A4	9561
Aspergillus niger CBS 513.88	10593
Aspergillus nomius NRRL 13137	11904
Aspergillus oryzae RIB40	12818
Aspergillus terreus NIH2624	10401
Aureobasidium namibiae CBS 147.97	10259
Aureobasidium subglaciale EXF-2481	10792
Auricularia subglabra TFB-10046 SS5	23555
Babjeviella inositovora NRRL Y-12698	6399
Batrachochytrium dendrobatidis JAM81	8700
Baudoinia panamericana UAMH 10762	10508
Beauveria bassiana ARSEF 2860	10364
Bipolaris maydis ATCC 48331	12705
Bipolaris oryzae ATCC 44560	12002
Bipolaris sorokiniana ND90Pr	12214
Bipolaris victoriae FI3	12882
Bipolaris zeicola 26-R-13	12853
Blastomyces gilchristii SLH14081	9587

Botrytis cinerea B05.10	16389
Candida albicans SC5314	6043
Candida auris	7461
Candida dubliniensis CD36	5859
Candida glabrata	5202
Candida orthopsilosis Co 90-125	5678
Candida tanzawaensis NRRL Y-17324	5885
Candida tenuis ATCC 10573	6985
Candida tropicalis MYA-3404	6254
Capronia coronata CBS 617.96	9231
Capronia epimyces CBS 606.96	10469
Chaetomium globosum CBS 148.51	11048
Chaetomium thermophilum var. *thermophilum* DSM 1495	7180
Cladophialophora bantiana CBS 173.52	12762
Cladophialophora carrionii CBS 160.54	10373
Cladophialophora immunda	14033
Cladophialophora psammophila CBS 110553	13421
Cladophialophora yegresii CBS 114405	10118
Clavispora lusitaniae ATCC 42720	5936
Coccidioides immitis RS	9910
Coccidioides posadasii C735 delta SOWgp	7229
Colletotrichum fioriniae PJ7	13759
Colletotrichum gloeosporioides Nara gc5	15381
Colletotrichum graminicola M1.001	12020
Colletotrichum higginsianum IMI 349063	14650
Colletotrichum orchidophilum	14453
Coniophora puteana RWD-64-598 SS2	13758
Coniosporium apollinis CBS 100218	9308
Coprinopsis cinerea okayama7#130	13356
Cordyceps militaris CM01	9651
Cryptococcus amylolentus CBS 6039	10306
Cryptococcus gattii WM276	6561
Cryptococcus neoformans var. *grubii* H99	7826
Cryptococcus neoformans var. *neoformans* B-3501A	6578
Cryptococcus neoformans var. *neoformans* JEC21	6594

Cutaneotrichosporon oleaginosum	8320
Cyberlindnera jadinii NRRL Y-1542	6032
Cyphellophora europaea CBS 101466	11094
Dactylellina haptotyla CBS 200.50	10959
Debaryomyces fabryi	6027
Debaryomyces hansenii CBS767	6272
Dichomitus squalens LYAD-421 SS1	12287
Diplodia corticola	10839
Encephalitozoon cuniculi GB-M1	1996
Encephalitozoon hellem ATCC 50504	1847
Encephalitozoon intestinalis ATCC 50506	1938
Encephalitozoon romaleae SJ-2008	1831
Endocarpon pusillum Z07020	9238
Enterocytozoon bieneusi H348	3632
Eremothecium cymbalariae DBVPG#7215	4432
Eremothecium gossypii ATCC 10895	4776
Eremothecium sinecaudum	4536
Eutypa lata UCREL1	11685
Exophiala aquamarina CBS 119918	13118
Exophiala dermatitidis NIH/UT8656	9578
Exophiala mesophila	10347
Exophiala oligosperma	13234
Exophiala spinifera	12049
Exophiala xenobiotica	13187
Fibroporia radiculosa	9262
Fomitiporia mediterranea MF3/22	11338
Fonsecaea erecta	12090
Fonsecaea monophora	11984
Fonsecaea multimorphosa CBS 102226	12369
Fonsecaea nubica	11681
Fonsecaea pedrosoi CBS 271.37	12527
Fusarium graminearum PH-1	13313
Fusarium oxysporum f. sp. *lycopersici* 4287	27347
Fusarium pseudograminearum CS3096	12397
Fusarium verticillioides 7600	20553

Gaeumannomyces tritici R3-111a-1	14650
Glarea lozoyensis ATCC 20868	13083
Gloeophyllum trabeum ATCC 11539	11755
Grosmannia clavigera kw1407	8312
Heterobasidion irregulare TC 32-1	13275
Histoplasma capsulatum NAm1	9313
Hyphopichia burtonii NRRL Y-1933	5996
Isaria fumosorosea ARSEF 2679	10061
Kalmanozyma brasiliensis GHG001	5765
Kazachstania africana CBS 2517	5375
Kazachstania naganishii CBS 8797	5319
Kluyveromyces lactis	5085
Kluyveromyces marxianus DMKU3-1042	4952
Kockovaella imperatae	7392
Komagataella phaffii GS115	5040
Kuraishia capsulata CBS 1993	5989
Kwoniella bestiolae CBS 10118	9133
Kwoniella dejecticola CBS 10117	8602
Kwoniella mangroviensis CBS 8507	8422
Kwoniella pini CBS 10737	7829
Laccaria bicolor S238N-H82	18215
Lachancea lanzarotensis	5056
Lachancea thermotolerans CBS 6340	5092
Leptosphaeria maculans JN3	12469
Lobosporangium transversale	11822
Lodderomyces elongisporus NRRL YB-4239	5799
Magnaporthe oryzae 70-15	12989
Malassezia globosa CBS 7966	4286
Malassezia pachydermatis	4202
Malassezia sympodialis ATCC 42132	3318
Marssonina brunnea f. sp. '*multigermtubi*' MB_m1	10027
Melampsora larici-populina 98AG31	16372
Metarhizium acridum CQMa 102	9849
Metarhizium brunneum ARSEF 3297	10689
Metarhizium majus ARSEF 297	11535

Metarhizium robertsii ARSEF 23	11688
Metschnikowia bicuspidata var. *bicuspidata* NRRL YB-4993	5838
Meyerozyma guilliermondii ATCC 6260	5920
Mitosporidium daphniae	3330
Mixia osmundae IAM 14324	6858
Moesziomyces antarcticus	6766
Moniliophthora roreri MCA 2997	17910
Nannizzia gypsea CBS 118893	8921
Naumovozyma castellii CBS 4309	5589
Naumovozyma dairenensis CBS 421	5546
Nectria haematococca mpVI 77-13-4	15708
Nematocida parisii ERTm1	2661
Neofusicoccum parvum UCRNP2	10366
Neurospora crassa OR74A	10812
Neurospora tetrasperma FGSC 2508	10380
Nosema ceranae BRL01	2060
Ogataea parapolymorpha DL-1	5325
Ogataea polymorpha	5173
Ordospora colligata OC4	1820
Paracoccidioides brasiliensis Pb18	8390
Paracoccidioides lutzii Pb01	8826
Paraphaeosphaeria sporulosa	14734
Parastagonospora nodorum SN15	15994
Penicilliopsis zonata CBS 506.65	9870
Penicillium arizonense	12200
Penicillium digitatum Pd1	8946
Penicillium expansum	11060
Penicillium rubens Wisconsin 54-1255	12791
Pestalotiopsis fici W106-1	15413
Phaeoacremonium minimum UCRPA7	8834
Phanerochaete carnosa HHB-10118-sp	13925
Phialocephala scopiformis	18567
Phialophora attae	11848
Phycomyces blakesleeanus NRRL 1555	16543
Pichia kudriavzevii	5385

Pichia membranifaciens NRRL Y-2026	5542
Pneumocystis carinii B80	3646
Pneumocystis jirovecii RU7	3761
Pneumocystis murina B123	3623
Pochonia chlamydosporia 170	14204
Podospora anserina S mat+	10548
Postia placenta Mad-698-R	9083
Pseudocercospora fijiensis CIRAD86	13066
Pseudogymnoascus destructans 20631-21	9153
Pseudogymnoascus verrucosus	10573
Pseudozyma hubeiensis SY62	7472
Puccinia graminis f. sp. *tritici* CRL 75-36-700-3	15979
Punctularia strigosozonata HHB-11173 SS5	11540
Purpureocillium lilacinum	11763
Pyrenophora teres f. *teres* 0-1	11799
Pyrenophora tritici-repentis Pt-1C-BFP	12169
Rasamsonia emersonii CBS 393.64	9843
Rhinocladiella mackenziei CBS 650.93	11382
Rhodotorula graminis WP1	7278
Rhodotorula toruloides NP11	8140
Saccharomyces cerevisiae S288C	6002
Saccharomyces eubayanus	5377
Saitoella complicata NRRL Y-17804	7034
Scedosporium apiospermum	8375
Scheffersomyces stipitis CBS 6054	5818
Schizophyllum commune H4-8	13194
Schizosaccharomyces cryophilus OY26	5180
Schizosaccharomyces japonicus yFS275	4878
Schizosaccharomyces octosporus yFS286	4986
Schizosaccharomyces pombe	5132
Sclerotinia sclerotiorum 1980 UF-70	14490
Serpula lacrymans var. *lacrymans* S7.9	12925
Setosphaeria turcica Et28A	11698
Sordaria macrospora k-hell	10827
Spathaspora passalidarum NRRL Y-27907	5983

Sphaerulina musiva SO2202	10156
Spizellomyces punctatus DAOM BR117	9422
Sporothrix schenckii 1099-18	10293
Stereum hirsutum FP-91666 SS1	14066
Sugiyamaella lignohabitans	5135
Talaromyces atroroseus	9523
Talaromyces marneffei ATCC 18224	10638
Talaromyces stipitatus ATCC 10500	13252
Tetrapisispora blattae CBS 6284	5388
Tetrapisispora phaffii CBS 4417	5252
Thermothelomyces thermophila ATCC 42464	9097
Thielavia terrestris NRRL 8126	9802
Tilletiaria anomala UBC 951	6808
Torulaspora delbrueckii	4978
Trametes versicolor FP-101664 SS1	14302
Tremella mesenterica DSM 1558	8308
Trichoderma atroviride IMI 206040	11816
Trichoderma gamsii	10961
Trichoderma reesei QM6a	9115
Trichoderma virens Gv29-8	12406
Trichophyton benhamiae CBS 112371	7974
Trichophyton rubrum CBS 118892	8706
Trichophyton verrucosum HKI 0517	8028
Trichosporon asahii var. *asahii* CBS 2479	8311
Tsuchiyaea wingfieldii CBS 7118	8094
Tuber melanosporum Mel28	7496
Uncinocarpus reesii 1704	7760
Ustilago maydis 521	6783
Vanderwaltozyma polyspora DSM 70294	5367
Vavraia culicis subsp. *floridensis*	2773
Verruconis gallopava	11357
Verticillium alfalfae VaMs.102	10237
Verticillium dahliae VdLs.17	10535
Vittaforma corneae ATCC 50505	2239
Wallemia ichthyophaga EXF-994	4863

Wallemia mellicola CBS 633.66	5277
Wickerhamomyces anomalus NRRL Y-366-8	6421
Wickerhamomyces ciferrii	6702
Xylona heveae TC161	8201
Yarrowia lipolytica CLIB122	6448
Zygosaccharomyces rouxii	4991
Zymoseptoria tritici IPO323	10963
