# Supplementary figures and images for: Gcorn fungi: A Web Tool for Detecting Biases between Gene Evolution and Speciation in Fungi
Source: J Fungi (Basel). 2021 Nov 12;7(11):959. doi: 10.3390/jof7110959 (PMC8624827; doi:10.3390/jof7110959)

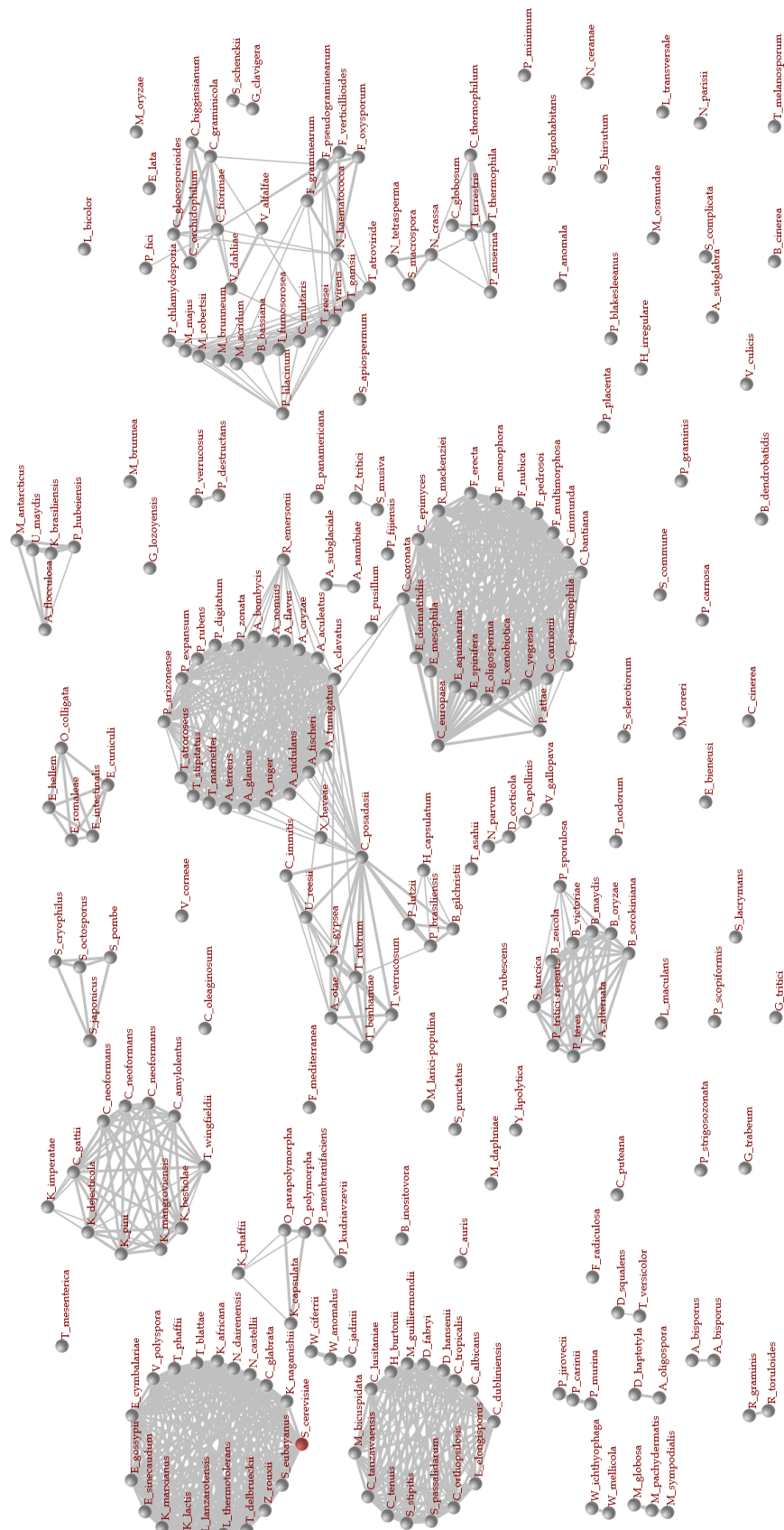

Supplement: Supplementary file 1 [file jof-07-00959-s001.zip › FigureS2a.pdf]

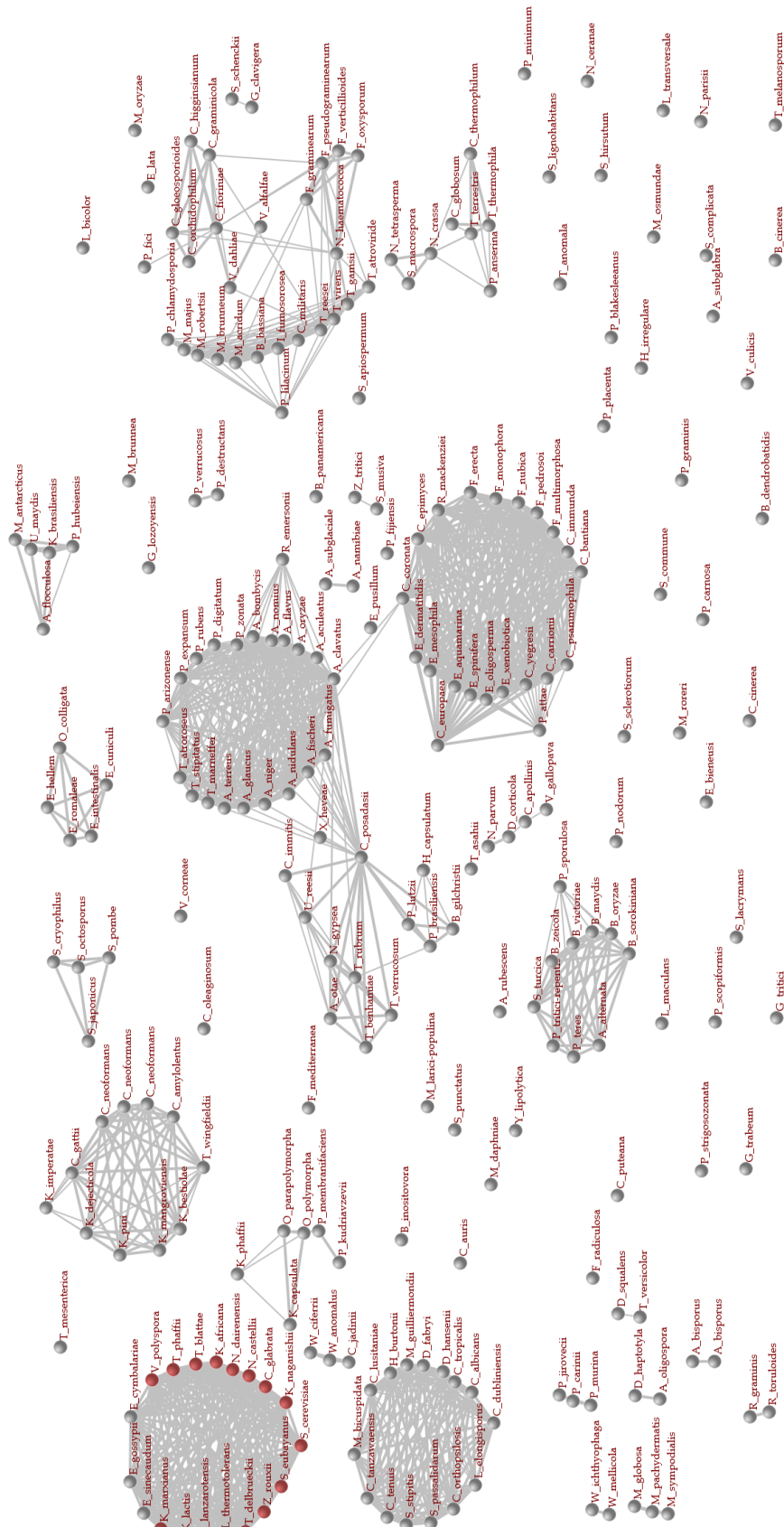

Supplement: Supplementary file 1 [file jof-07-00959-s001.zip › FigureS2b.pdf]

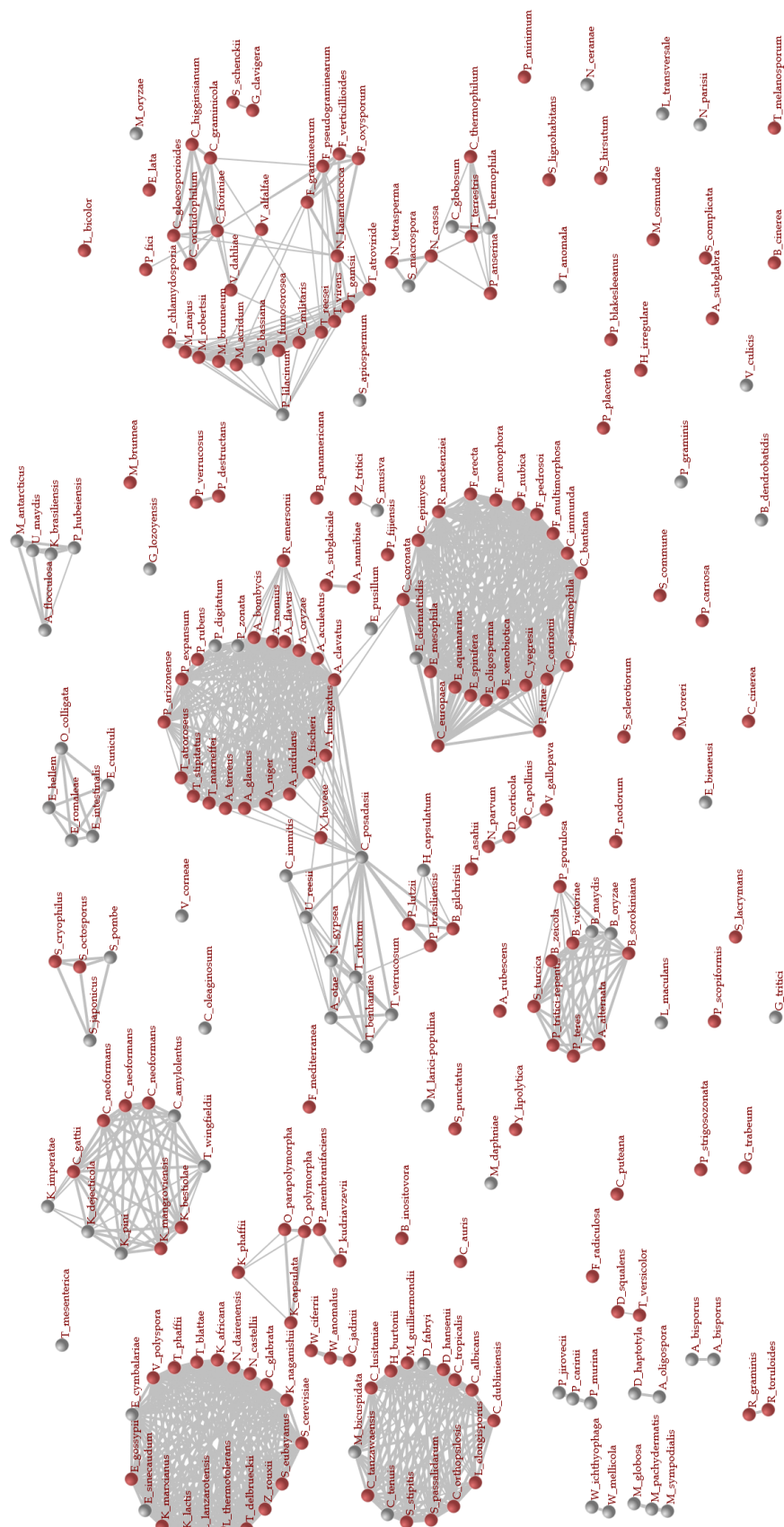

Supplement: Supplementary file 1 [file jof-07-00959-s001.zip › FigureS2c.pdf]

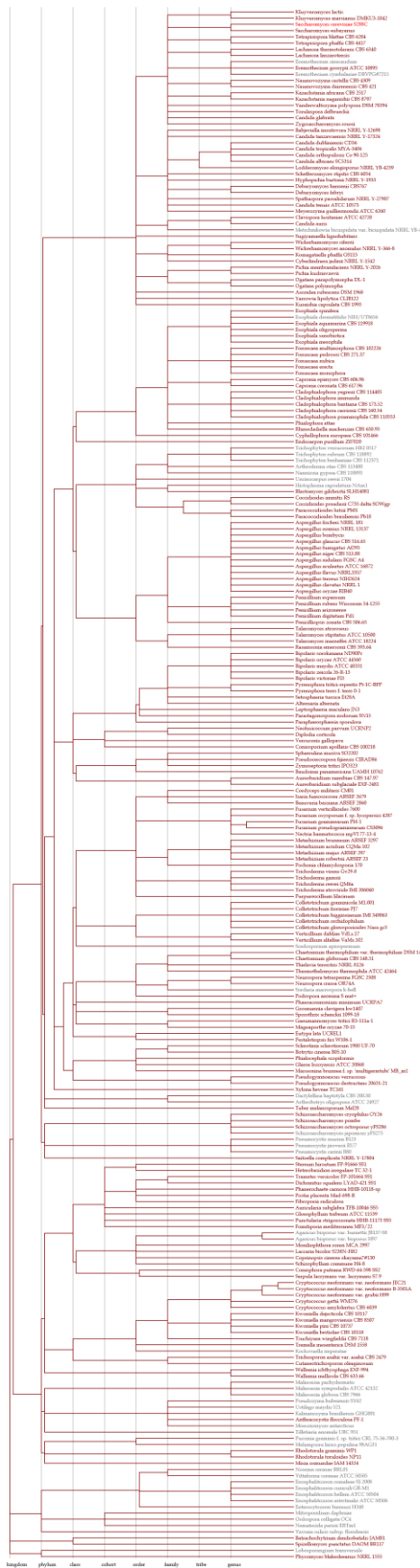

Supplement: Supplementary file 1 [file jof-07-00959-s001.zip › FigureS3a.pdf]

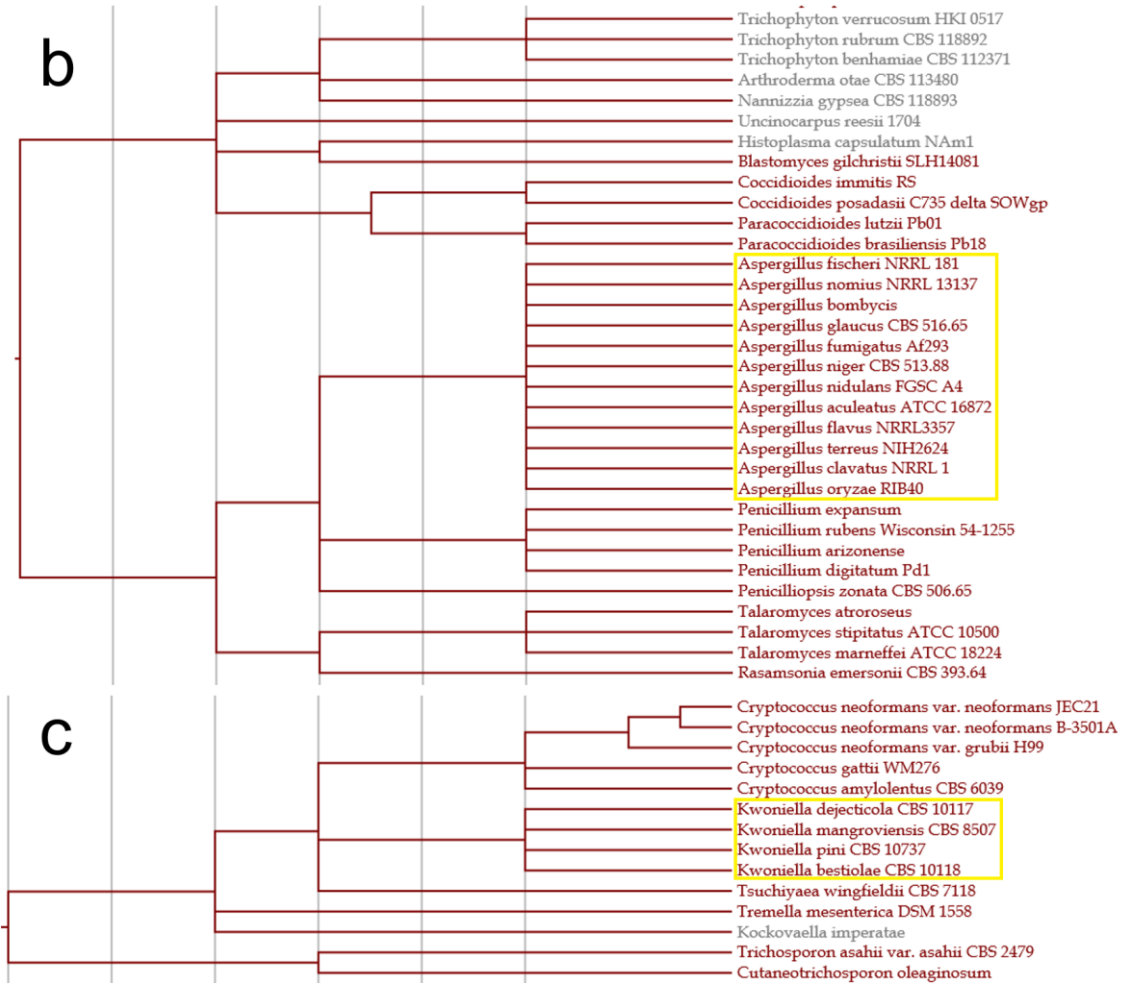

Supplement: Supplementary file 1 [file jof-07-00959-s001.zip › FigureS3bc.pdf]

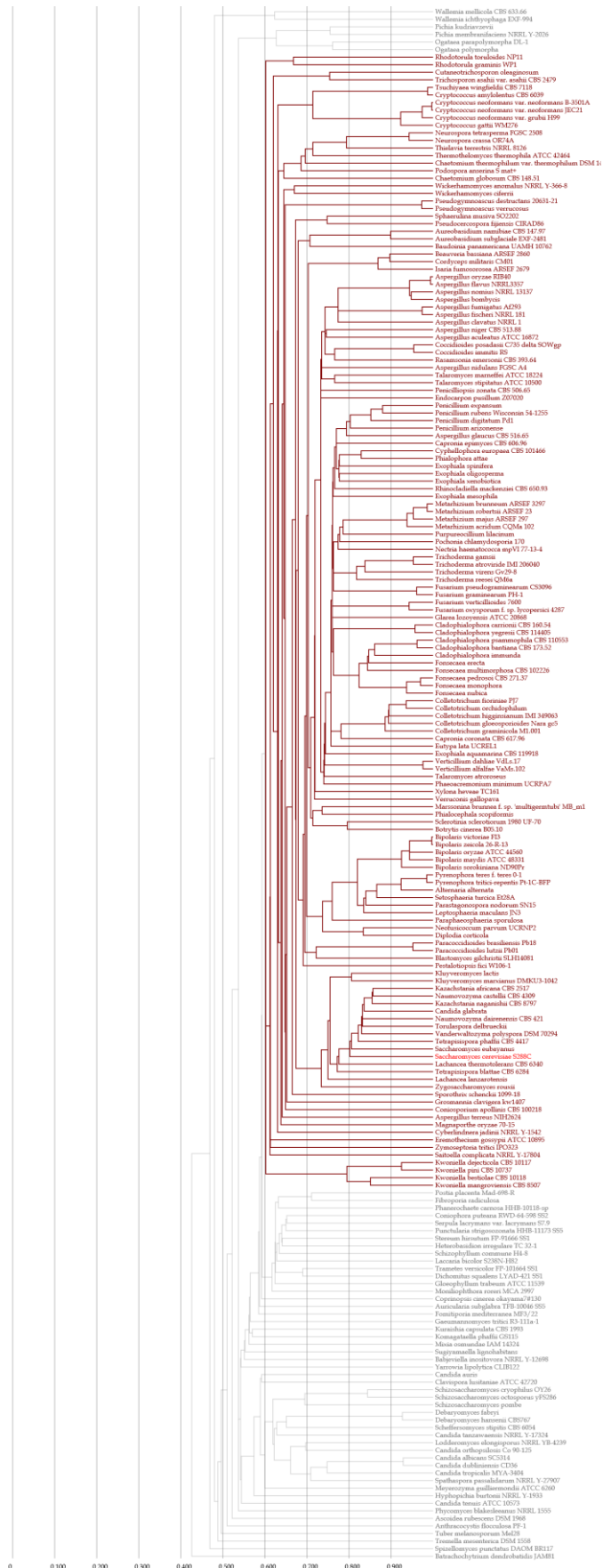

Supplement: Supplementary file 1 [file jof-07-00959-s001.zip › FigureS4a.pdf]
